# Inflammatory bowel disease is associated with an increased risk of cardiovascular events in a sex and age-dependent manner: A historical cohort study

**DOI:** 10.1016/j.ijcrp.2025.200363

**Published:** 2025-01-04

**Authors:** Noa Cohen-Heyman, Gabriel Chodick

**Affiliations:** a Department of Epidemiology and Preventive Medicine, School of Public Health, Faculty of Medicine, Tel Aviv University, Ramat Aviv, Tel Aviv, 6997801, Israel; b Department of Internal Medicine T, Sheba Medical Center, Tel Hashomer, Ramat Gan, Israel; c Maccabi Institute for Research and Innovation, Maccabi Healthcare Services, Tel Aviv, 68125, Israel

**Keywords:** Inflammatory bowel disease, Crohn's disease, Ulcerative colitis, Ischemic heart diseases, Inflammation, Males

## Abstract

**Background:**

The relationship between inflammatory bowel diseases (IBD) and the risk of ischemic heart diseases (IHD) remains a subject of debate. In this study, we sought to investigate the association between IBD and long-term risk of IHD in a substantial cohort of IBD patients.

**Methods:**

In this retrospective cohort study we utilized data from a state-mandated provider in Israel (Maccabi Healthcare Services). We identified all eligible patients diagnosed with IBD between 1/1990 and 7/2021 that were individually matched by sex-and-birth date to 10 MHS members with no indication of IBD. Study population was followed through the data until 12/2021 to examine the occurrence of IHD events.

**Results:**

A total of 14,768 IBD patients (6144 UC, 8624 CD) and 120338 matched non-IBD individuals were eligible for the analysis. Over a mean follow-up of 10.5 years, 285 (1.9 %) of participants with IBD and 1175 (1.0 %) of the reference group experienced our composite outcome, representing an HR of 1.98 (95%CI: 1.74–2.25). When stratified by sex, risk of IHD associated with IBD in males (HR = 1.82; 95 % CI: 1.52–2.17), whereas a negative association was noted among female patients (HR = 0.72; 95%CI: 0.55–0.95). Study results were generally unchanged when analyses were limited to patients with CD, UC, patients on steroids, and patients on immunosuppressants.

**Conclusions:**

Our study reveals a notable excess risk of IHD in male patients with IBD. Further research is needed to better elucidate the mechanisms involved in this relationship.

## 1 Introduction

Inflammatory bowel disease (IBD), once perceived as primarily afflicting the Western world, has emerged as a global health concern, imposing a substantial burden of morbidity [1]. The underlying mechanism of the chronic inflammation characteristic of IBD manifests through autoimmune dysfunction within the gastrointestinal tract immune system [2]. This immune dysregulation leads to the release of various mediators and cytokines into the bloodstream, including tumor necrosis factor-alpha (TNF-α), nitric oxide, interleukin 6, and liver proteins such as C-reactive protein (CRP), culminating in endothelial cell dysfunction [3]. The compromised intestinal mucosal barrier in individuals with IBD allows microbial lipopolysaccharides (LPS) to initiate pro-inflammatory innate responses via Toll-like receptors (TLRs), ultimately resulting in endothelial damage and atherosclerosis [[4], [5], [6]].

Studies investigating endothelial function in IBD patients have revealed diminished pulse arterial tonometry and shear stress reactive hyperemia, indicative of impaired blood vessel dilation in response to induced ischemia [7]. Furthermore, IBD is associated with reduced coronary flow reserve (CFR), and this reduction in CFR is closely linked to elevated high-sensitivity CRP levels [8]. The injured endothelial cells promote turbulent blood flow, creating vulnerable areas along the vascular walls, thereby instigating the atherosclerotic process and fostering the formation of blood clots ((9. Additionally, IBD has been linked to aortic stiffness, a vascular biomarker and independent predictor of cardiovascular events, even when accounting for established risk factors, as recently reported in numerous studies [[9], [10], [11], [12], [13]].

The shared pathological mechanisms between IBD and atherosclerosis have prompted inquiries into a potential association between IBD and an elevated risk of stroke and ischemic heart diseases (IHD) [14]. A meta-analysis encompassing data from 10 cohort studies reported an adjusted pooled relative risk (RR) of 1.24 (95 % CI, 1.14–1.35) for IHD among individuals with IBD. Sub-analyses revealed higher RRs among patients with Crohn's disease (CD), females, and those aged over 50 years [15]. However, some of the included studies lacked comprehensive information on potentially critical confounders such as tobacco use, obesity, and the history of IBD therapies. More recent investigations and meta-analyses, incorporating extended follow-up periods and multivariate adjustments, have disclosed a pooled elevated risk of myocardial infarction (MI) for both CD and ulcerative colitis (UC) with hazard ratios (HRs) of 1.36 (95 % CI, 1.12–1.64) for CD and 1.24 (95 % CI, 1.05–1.46) for UC, as well as for other cardiovascular diseases, including stroke, with HRs of 1.22 (95 % CI, 1.01–1.49) for CD and 1.09 (95 % CI, 1.03–1.15) for UC [16,17]. Nevertheless, individual studies within this meta-analysis, as well as additional research, have yielded inconsistent or inconclusive findings concerning the risk differential among IBD subgroups, age groups, sexes, types of IBD treatments, and other host and disease-related factors.

Given the limitations of existing data and the absence of specific preventive programs targeting cardiovascular diseases in IBD patients, we embarked on the present study to investigate the potential association between IBD and IHD within a substantial cohort of Israeli patients living with IBD.

## 2 Methods

### 2.1 Study population

This retrospective cohort study was performed using computerized databases of Maccabi Healthcare Services (MHS), a state-mandated provider of 2.6 million Israeli members throughout the country. Anonymized data were retrieved via MDClone data analytics environment. Eligible patients were MHS members that were included in the MHS's IBD registry [18] between January 1990 and July 2021. The date of IBD diagnosis was defined as index date. Excluded were those aged under 18 or over 95 years on the index date, had a follow-up period shorter than 180 days, had a documented history of IHD, chronic kidney disease, or hypercoagulability prior to index date. These patients were individually matched by birth date and sex to MHS members with no indication of IBD at a ratio of 1:10, after applying the same exclusion criteria as in the IBD cohort. To prevent surveillance bias and ensure that individuals in the reference have an equal opportunity to access care and be examined by a primary care physician (PCP), eligible patients were required to have at least one PCP visit during the study period. To increase the specificity of our definition for incident IHD, we further excluded patients treated with statins or acetylsalicylic acid (ASA) prior to index date.

IHD events occurring during the study follow-up period since IBD diagnosis were collected from MHS's CVD registry [19] and included the earliest of the following diseases/conditions: IHD, myocardial infarction (MI), as well as undergoing percutaneous coronary intervention (PCI) or coronary artery bypass grafting (CABG). Details for each outcome are provided in Appendix Table S1. MHS's CVD registry integrates personal records from clinics and hospitals, lab results, procedure reports, physical examinations, medications and radiologic tests.

### 2.2 Other study variables

Demographics and clinical data were extracted from the personal electronic health records, including tobacco smoking (past, current, or never smoker). We collected data on underlying conditions as hypertension, hypercholesterolemia, pre-diabetes and diabetes that were defined based on a physician's diagnosis and according to MHS registries. Body mass index (BMI) was calculated as weight in kilograms divided by height in meter square and regularly taken medications were included if dispensed from pharmacy.

### 2.3 Statistical analysis

Population characteristics were examined by study groups, using Chi-square test for categorical variables and *t*-test for continues variables. We considered p value < 0.05 as significant difference. To assess effect size, we calculated standard mean difference (SMD) and its 95%CI. We considered absolute SMD larger than 0.5 to be meaningful [20].

The incidence density per 100,000 person years (PY) of CVD composite outcome was calculated in terms of person-day unit, which is the actual number of days that individuals are at risk of IHDs. We summed the days of observation which started from the index date to the earliest date of IHD event, leaving MHS, death, or end of 2021. For survival analysis, the Kaplan–Meier curve for IHDs was plotted, and the log-rank test was calculated to compare the risk of IHD curve between males and females. We used univariate and multivariable Cox regression models to investigate the relationships between additional predictors and the IHD outcome, and expressed the results as hazard ratios (HRs) and 95 % confidence intervals (CI). To assess effect modification, we stratified the analyses by age, sex, type, and severity of IBD as indicated by therapy classes. We examined a quadratic relationship between age-and-sec specific HR for IHD and IBD status using formula y ∼ poly(x, 2) and default smoothing parameters using the function ‘geomsmooth’ in the R package ‘ggplot2’ version 3.4.2. Age at baseline was explored as a potential effect modifier by analyzing its interaction with IBD status. We used multiple imputations method to replace missing values for smoking status and BMI. The IBM-SPSS Statistical Package version 27.0 and R 4.3.1 software [21] were used to perform all statistical analyses.

### 2.4 Ethical considerations

The study has been conducted in accordance with ethical standards, and we have obtained approval from the Maccabi Healthcare Services' institutional review board.

## 3 Results

Our study included a total of 135,106 participants comprising 14,768 patients with IBD (6144 UC, 8624 CD) and 120,338 matched non-IBD individuals (Fig. S1). Patients with IBD were somewhat more likely to be older, males, and to have hypertension compared to the reference group (Table 1). None of these differences were considered strong.

**Table 1:** Study population characteristics.

Characteristics	IBD			Non-IBD	SMD (95 % CI)	P for UC vs. CD
	**AII (n = 14,768)**	**UC (n = 6144)**	**CD (n = 8624)**	**(n = 120,338)**		
	**n (%)**	**n (%)**	**n (%)**	**n (%)**		
Sex, males	7351 (49.8)	2947 (48.0)	4404 (51.1)	49227 (40.9)	0.20 (0.18,0.22)	**<0.01**
Age, y mean (SD)	44.55 (15.9)	46.94 (16.4)	42.85 (15.3)	40.70 (13.8)	0.27 (0.25,0.29)	**<0.01**
Smoking, ever	1568 (13.8)	627 (13.3)	941 (14.2)	13439 (14.7)	−0.04 (−0.06,-0.01)	**0.149**
BMI, kg/m^−2^ mean (SD)	24.09 (4.8)	24.45 (4.7)	23.84 (4.9)	25.00 (5.3)	−0.17 (−0.19,-0.15)	**<0.01**
Diabetes	125 (0.8)	77 (1.3)	48 (0.6)	736 (0.6)	0.15 (0.05,0.26)	**<0.01**
Hypertension	799 (5.4)	357 (5.8)	442 (5.1)	13803 (11.5)	−0.45 (−0.49,-0.41)	**0.087**
Hypercholesterolemia	775 (5.2)	353 (5.7)	422 (4.9)	10930 (9.1)	−0.33 (−0.37,-0.29)	**0.017**
**IBD therapy**						
Steroids	5226 (35)	2136 (34)	3090 (36)			**0.063**
Immunosuppressant	5825 (39)	1604 (26)	4221 (49)			**<0.01**
Any IBD therapy	8893 (61)	3122 (52)	5771 (67)			**<0.01**
Untreated for IBD	5743 (39)	2962 (48)	2781 (33)			**<0.01**

IBD, IBD, inflammatory bowel disease; UC, ulcerative colitis; CD, Crohn's disease; SMD, standard mean difference, calculated for all for all IBD vs non-IBD.BMI, body mass index; CVD, cardiovascular diseases. # All p-values were <0.001.

Table 2 depicts the frequency of IHD events for patients with IBD and their matched reference group. During a mean retrospective follow-up of 10.5 years, the cumulative incidence rates of IHD among patients with IBD, UC, CD, and reference group were 1.9 % (n = 285), 2.4 % (n = 145) and 1.6 % (n = 140), and 1.0 % (n = 1175), respectively.

**Table 2:** Incidence density (per 100,000 PY) and hazard ratio for ischemic heart diseases by IBD status and therapy groups.

	CVD cases	Incidence density per 100,000 PY (95 % CI)	HR (95 % CI)
IBD status			
**None**	1172	98 (0.93–104)	1 (ref.)
**All IBD**	285	178 (159–200)	1.98 (1.74–2.25)
**CD**	140	152 (129–179)	1.55 (1.31–1.85)
**UC**	145	216 (184–255)	2.31 (1.95–2.74)
IBD therapy			
**Steroids**	144	238 (195–291)	2.80 (2.37–3.33)
**Immunosuppressant**	44	126 (094–169)	1.59 (1.28–1.96)
**Any IBD therapy**	188	180 (156–208)	2.15 (1.85–2.50)
**Untreated for IBD**	97	177 (145–215)	1.71 (1.39–2.10)

IBD, inflammatory bowel disease; UC, ulcerative colitis; CD, Crohn's disease; CVD, cardiovascular diseases; IR, incidence; HR, crude hazard ratio.

Significant differences in IHD risk were found between the IBD groups (Table 2 and Fig. S2). Patients with IBD had an HR of 1.98 (95%CI: 1.74–2.25) for CVD compared to the reference group with an effect size of approximately 800 per 100,000PY. Substantial difference in IHD occurrence were also recorded among IBD patients with the highest HR calculated among patients treated with steroids (2.80; 95%CI: 2.37–3.33)) and the lowest in patients treated with immunosuppressant drugs (1.59; 95%CI: 1.28–1.96).

**Fig. 1:**
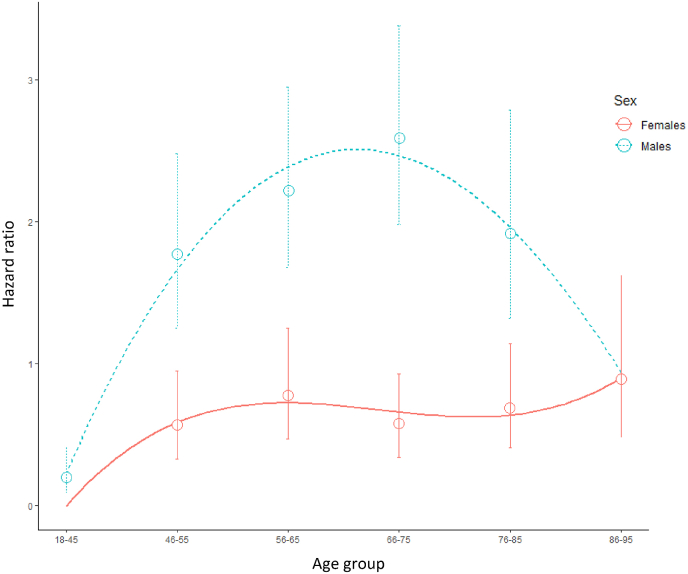
Age-and-sex specific hazard ratio (HR) and 95 % confidence intervals for ischemic heart diseases among IBD and individually matched non-IBD patients. Blue dotted line curve stands for males HR; blue dotted vertical lines represent 95 % confidence interval of HR. Red continuous line curve stands for females HR; red dotted vertical lines represent 95 % confidence interval of HR.

When stratified by sex, risk of IHD was associated with IBD in males (HR = 1.82; 95 % CI: 1.52–2.17), whereas a negative association was noted among female patients (HR = 0.72; 95%CI: 0.55–0.95). In both sexes there was a significantly (p < 0.001) positive interaction between age and IBD with regards to IHD risk. Age-and-sex specific Kaplan-Meier curves and their 95 % CI of new onset IHD among the study groups is given in Supplementary Fig. S3. In multivariable analysis, IBD was associated with an increased risk of IHD among males in all age groups, between 46 and 85 years, but not among females. Moreover, while the HR for IHD among males aged 46–55 with IBD was 1.77 (95%CI: 1.25–2.48) compared to males in the reference group, females with IBD in this age group had a decreased risk of IHD compared with non-IBD females in the same age group (HR = 0.57, 95 % CI:0.33–0.95) (Fig. 1 and Table S3).

**Fig. 2:**
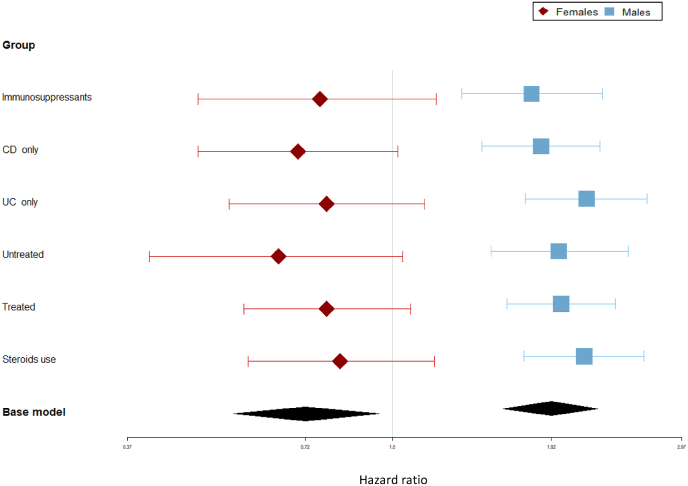
Adjusted HR∗ of IHD for all IBD patients and according to IBD subgroups and therapy after stratification according to sex. Blue horizontal line and rectangle stands for males HR and 95 % confidence interval. Red horizontal line and rhombus stands for females HR and 95 % confidence interval. Black rhombus (“base model”) stands for HR and 95 % confidence interval for “all IBD”. Adjusted HR∗ for age, hypertension, hyperlipidemia, diabetes mellitus and BMI.

The increased risk for IHD in males with IBD remained significant after adjusting for baseline age, hypertension, hyperlipidemia, T2DM, and BMI, with comparable HRs of 1.82 (95%CI:1.52–2.17), 1.75 (95%CI:1.40–2.19) and 2.08 (95%CI:1.65–2.61) in patients with IBD, CD, and UC compared to reference group, respectively (Fig. 2 and Table S3). No indication of increased IHD was found among female groups and the reduced adjusted HRs remained significant only in the base model.

## 4 Discussion

The etiology of IBD as an immune-mediated condition has spurred inquiries into its potential association with an increased risk of IHD(14). In this retrospective cohort analysis, we found a clinically-relevant increased risk of IHD among male adults with IBD, even after adjusting for age and a spectrum of traditional risk factors. This consistent risk pattern extended to patients with UC, CD, and across various IBD treatment strategies, with the highest adjusted hazard ratio observed in patients diagnosed with UC (2.08; 95 % CI: 1.65–2.61).

Notably, the risk estimates for IHD in our IBD cohort exceeded the pooled HR (1.50; 95%CI 1.16 to 1.92) of acute coronary syndrome reported in a recent meta-analysis encompassing 12 studies and 225,248 patients with IBD [22]. This is despite the similarity between our cohort and the studies included in the meta-analysis in age, sex, and smoking rates as well as in CD to UC ratio.

One significant discovery in our study is the significant sex and age-dependent association between IBD and an increased risk of IHD. Previous research has revealed higher CRP levels in females with chronic inflammatory diseases [23]. Given that CRP is independently correlated with an elevated CVD risk, we initially anticipated that IBD would confer a greater IHD risk among females. Surprisingly, when stratified by sex and age, we found a 1.7–2.6-fold higher HR for IHD among older males with IBD while an opposite trend of negative association was observed in females reaching statistical significance for the age group 46–55. Our findings deviate from those summarized in a few meta-analyses that indicated a higher CVD risk among females with IBD compared to males, although these analyses often did not adequately adjust for potential confounders [22,24]. Our results align more closely with a recent large population-based historical prospective study utilizing UK-biobank data, which reported an adjusted HR of 1.87 (95 % CI: 1.52–2.31) specifically for males [17]. This disparity may be attributed, in part, to the reproductive years of females diagnosed with IBD, when pregnancy was associated with a decreased risk of IBD flares in the following years, as shown in a European cohort of patients with IBD [25]. Additionally, the lesser use of steroids during pregnancy, a known IHD risk factor, may also play a role [26].

Our study results indicate an age-effect of the association between IBD and IHD. The hazard ratio for IHD was most pronounced in patients aged of 66–75 years (with an absolute risk difference of 650 per 100,000 person-years). In line with a recently published national cohort study from Sweden [27] on 76,517 patients with IBD and risk of acute coronary syndrome, we found no indication for elevated risk among younger adults under the age of 45. This is particularly important in Israel where the IBD incidence rate of older adult patients is on the high end compared to Europe and the United States [28].

The role of chronic inflammation as a pivotal contributor to IHD is supported by previous studies conducted on systemic lupus erythematosus, rheumatoid arthritis, and psoriasis [29,30]. A comprehensive investigation encompassing 19 different inflammatory diseases, including IBD, reinforced this notion, demonstrating an increased risk with a hazard ratio of 1.7. Furthermore, it revealed that the risk escalated with the number of autoimmune diseases, with hazard ratios of 1.4, 2.6, and 3.79 for one, two, or three diagnoses, respectively [31]. A few randomized controlled trials have examined the effect of anti-inflammatory therapies, such as canakinumab and colchicine, to reduce the risk of CVD recurrence in patients with IHD, demonstrating risk reductions of 15 % and 30 %, respectively. These findings underscore the concept of inflammation as an independent risk factor for IHD [32,33].

The strength of our study primarily stems from its extensive cohort, representative of the population, and reliable, validated registries. The large study population allowed for stratification and comprehensive subgroup analyses. Additionally, there were minimal missing values for most covariates, exposure, and outcome variables, with imputation analysis supporting the original dataset-based results. However, our study does have limitations, including the absence of data on other IHD risk factors, such as physical activity and relevant family history, along with data on other inflammatory diseases linked to IBD, the presence of extraintestinal manifestations, and data on flare and biomarkers of disease activity. A thorough investigation of these factors in future studies could enhance our understanding of the association between IBD and IHD. Moreover, the IBD patient cohort is susceptible to surveillance bias due to close monitoring, which could lead to increased diagnoses of other comorbidities [34]. To mitigate this potential bias, we selected controls with other medical diagnoses, excluding inflammatory diseases. To ensure that individuals in the reference have an equal opportunity to access care and be examined by primary care physician (PCP), eligible patients were required to have at least one PCP visit during the study period. This may have increased the likelihood of non-IBD patient to have clinical conditions that may relate to IHD (e.g. hypertension). This was partially mitigated by excluding patients on statins or ASA prior to index date. Additionally, the nature of the studied outcome, cardiovascular disease, typically prompts patients to seek medical attention, making it less likely to be overlooked. Finally, the use of electronic medical records for research necessitates careful consideration of misclassification and measurement errors. In our study, we employed validated registries that have been widely used in previous research, both for the diagnosis of exposure (IBD) and the outcome (IHD), ensuring a robust and reliable database.

In conclusion, our study underscores the significant excess risk of IHD in individuals with IBD, particularly among males and older patients. It is increasingly evident that individuals with IBD should be closely monitored for additional risk factors that may interact synergistically, amplifying their likelihood of developing IHD. Encouraging a healthy lifestyle, including adherence to the Mediterranean diet and regular physical activity, should be a priority. Furthermore, risk stratification based on CRP levels, disease flares, and hospitalizations may help identify patients in need of vigilant monitoring. High-risk individuals could be evaluated for early biomarkers predictive of future IHD, such as high-sensitive cardiac troponin, potentially making them candidates for preventive interventions, including daily aspirin administration [[35], [36], [37]].

## CRediT authorship contribution statement

**Noa Cohen-Heyman:** Writing – original draft, Methodology, Formal analysis, Data curation, Conceptualization. **Gabriel Chodick:** Writing – review & editing, Supervision, Methodology, Conceptualization.

## Support sources

No grants or funding for this work.

## Data availability statement

NCH had full access to the data and take responsibility for the content.

## Declarations of interest

None.
