# Supplementary material for: Inflammatory bowel disease is associated with an increased risk of cardiovascular events in a sex and age-dependent manner: A historical cohort study
Source: Int J Cardiol Cardiovasc Risk Prev. 2025 Jan 4;24:200363. doi: 10.1016/j.ijcrp.2025.200363 (PMC11760278; doi:10.1016/j.ijcrp.2025.200363)
Supplement: Multimedia component 1 [file mmc1.docx]

**Supplementary material**

**Table S1: List of ICD-9 codes used to create the study outcomes.**

| ICD-9 diagnosis codes | |
| --- | --- |
| 410 | Acute Myocardial Infarction |
| 410.01 | Acute Myocardial Infarction Of Anterolateral Wall, Initial Episode Of Care |
| 410.02 | Acute Myocardial Infarction Of Anterolateral Wall, Subsequent Episode Of Care |
| 410.1 | Acute Myocardial Infarction Of Other Anterior Wall Infarction |
| 410.11 | Acute Myocardial Infarction Of Other Anterior Wall, Initial Episode Of Care Infarction |
| 410.12 | Acute Myocardial Infarction Of Other Anterior Wall, Subsequent Episode Of Care Infarction |
| 410.2 | Acute Myocardial Infarction Of Inferolateral Wall |
| 410.21 | Acute Myocardial Infarction Of Inferolateral Wall, Initial Episode Of Care |
| 410.22 | Acute Myocardial Infarction Of Inferolateral Wall, Subsequent Episode Of Care |
| 410.3 | Acute Myocardial Infarction Of Inferoposterior Wall |
| 410.31 | Acute Myocardial Infarction Of Inferoposterior Wall, Initial Episode Of Care |
| 410.32 | Acute Myocardial Infarction Of Inferoposterior Wall, Subsequent Episode Of Care |
| 410.4 | Acute Myocardial Infarction Of Other Inferior Wall Infarction: {diaphragmatic wall} |
| 410.41 | Acute Myocardial Infarction Of Other Inferior Wall, Initial Episode Of Care Infarction |
| 410.42 | Acute Myocardial Infarction Of Other Inferior Wall, Subsequent Episode Of Care Infarction |
| 410.5 | Acute Myocardial Infarction Of Other Lateral Wall |
| 410.51 | Acute Myocardial Infarction Of Other Lateral Wall, Initial Episode Of Care |
| 410.52 | Acute Myocardial Infarction Of Other Lateral Wall, Subsequent Episode Of Care |
| 410.6 | True Posterior Wall Infarction |
| 410.61 | True Posterior Wall Infarction, Initial Episode Of Care |
| 410.62 | True Posterior Wall Infarction, Subsequent Episode Of Care |
| 410.7 | Subendocardial Infarction |
| 410.71 | Subendocardial Infarction, Initial Episode Of Care |
| 410.72 | Subendocardial Infarction, Subsequent Episode Of Care |
| 410.8 | Acute Myocardial Infarction Of Other Specified Sites |
| 410.81 | Acute Myocardial Infarction Of Other Specified Sites, Initial Episode Of Care |
| 410.82 | Acute Myocardial Infarction Of Other Specified Sites, Subsequent Episode Of Care |
| 410.9 | Acute Myocardial Infarction Of Unspecified Site Acute myocardial infarction NOS; |
| 410.91 | Acute Myocardial Infarction Of Unspecified Site, Initial Episode Of Care Acute myocardial infarction NOS |
| 410.92 | Acute Myocardial Infarction Of Unspecified Site, Subsequent Episode Of Care Acute myocardial infarction NOS |
| 411 | Other Acute And Subacute Forms Of Ischemic Heart Disease |
| 411 | Post-myocardial Infarction Syndrome |
| 411.01 | Dressler's syndrome |
| 411.1 | Intermediate Coronary Syndrome Impending infarction; Preinfarction angina; Preinfarction syndrome; Unstable angina |
| 411.8 | Other Acute And Subacute Forms Of Ischemic Heart Disease |
| 411.81 | Other Acute And Subacute Forms Of Ischemic Heart Disease, Acute Ischemic Heart Disease Without Myocardial Infarction |
| 411.89 | Other Acute And Subacute Forms Of Ischemic Heart Disease, Other Coronary insufficiency (acute |
| 414 | Other Forms Of Chronic Ischemic Heart Disease |
| 414.0 | Coronary atherosclerosis |
| 414.00 | Coronary atherosclerosis of unspecified Type Of Vessel, Native Or Graft |
| 414.01 | Coronary atherosclerosis of native coronary vessel Atherosclerotic heart disease; Coronary (artery) |
| 414.02 | Coronary atherosclerosis of autologous vein bypass graft Atherosclerotic heart disease; Coronary |
| 414.03 | Coronary atherosclerosis of nonautologous biological bypass graft. Atherosclerotic heart disease; Coron |
| 414.04 | Coronary Atherosclerosis of Artery Bypass Graft Internal Mammary Artery |
| 414.05 | Coronary Atherosclerosis of Unspecified Type Of Bypass Graft Bypass Graft NOS |
| 414.8 | Other Specified Forms Of Chronic Ischemic Heart Disease |
| 414.9 | Chronic Ischemic Heart Disease, Unspecified Ischemic heart disease NOS |
| 429.2 | Cardiovascular Disease, Unspecified Arteriosclerotic cardiovascular disease [ASCVD] |
| 429.7 | Certain Sequelae Of Myocardial Infarction, NEC |
| 429.79 | Certain Sequelae Of Myocardial Infarction, NEC, Other Mural thrombus (atrial) (ventricular) acquired, following myocardial infarction |
| **ICD-9 procedure codes** | |
| 0.66 | Percutaneous Transluminal Coronary Angioplasty [ptca] Or Coronary Atherectomy |
| 36 | Operations On Vessels Of Heart |
| 36.0 | Removal Of Coronary Artery Obstruction And Insertion Of Stent(s) |
| 36.01 | Ptca W/out Thrombolyt Ag |
| 36.02 | Ptca With Thrombolyt Ag |
| 36.03 | Op Coron Art Angioplasty |
| 36.04 | In/cor Art Thromboly Inf |
| 36.05 | Multiple Vessel Ptca |
| 36.06 | Insertion Of Non-drug-eluting Coronary Artery Stent(S) |
| 36.07 | Insertion Of Drug-eluting Coronary Artery Stent(S) |
| 36.09 | Other Remo Cor Art Obst |
| 36.1 | Bypass Anastomosis For Heart Revascularization Billable |
| 36.1 | Aortocoronary Bypass Nos |
| 36.11 | Aortocoronary Bypass Of One Coronary Artery |
| 36.12 | Aortocoronary Bypass Of Two Coronary Arteries |
| 36.13 | Aortocoronary Bypass Of Three Coronary Arteries |
| 36.14 | Aortocoronary Bypass Of Four Or More Coronary Arteries |
| 36.15 | Single Internal Mammary-Coronary Artery Bypass |
| 36.16 | Double Internal Mammary-Coronary Artery Bypass |
| 36.19 | Other Bypass Anastomosis For Heart Revascularization |
| 36.2 | Heart Revascularization By Arterial Implant |
| 36.3 | Other Heart Revascularization |
| 36.9 | Other Operations On Vessels Of Heart |

**Table S2: Age-and-sex specific incidence density of CVD events among IBD and individually matched reference group**

|  | CVD risk (ID per 100,000 PY) | | | | HR (95% CI) | HR (95% CI) |  |
| --- | --- | --- | --- | --- | --- | --- | --- |
| Age group (years) | IBD | | Reference group | |  |  |  |
|  | **Females** | **Males** | **Females** | **Males** | **Females** | **Males** |  |
| **18-45 (n=84872)** | 0 | 5 (14) | 208 (52) | 160 (70) | 0 | 0.20 (0.09-0.41) |  |
| **46-55 (n=29415)** | 10 (46) | 32 (156) | 216 (81) | 84 (88) | 0.57 (0.33-0.95) | 1.77 (1.25-2.48) |  |
| **56-65 (n=13152)** | 13 (103) | 53 (407) | 130 (132) | 90 (183) | 0.78 (0.47-1.25) | 2.22 (1.68-2.95) |  |
| **66-75 (n=5542)** | 13 (161) | 76 (1060) | 77 (280) | 77 (410) | 0.58 (0.34-0.93) | 2.59 (1.98-3.38) |  |
| **76-85 (n=1528)** | 15 (435) | 38 (1582) | 37 (629) | 40 (826) | 0.69 (0.41-1.14) | 1.92 (1.32-2.79) |  |
| **86-95 (n=464)** | 11 (1210) | 19 (2436) | 24 (1355) | 29 (2587) | 0.89 (0.48-1.62) | 0.94 (0.57-1.53) |  |
| **IBD, inflammatory bowel disease; CVD, cardiovascular diseases; PY, person years; HR, crude hazard ratio; ID, incidence density** | | | | | | | |

**Table S3: Adjusted hazard ratios (aHR) for CVD by sex and IBD status and therapy group**

|  | aHR (95% CI) | |
| --- | --- | --- |
|  | **Females** | **Males** |
| IBD status |  |  |
| **None** | 1 (ref) | 1 (ref) |
| **All IBD** | 0.72 (0.55-0.95) | 1.82 (1.52-2.17) |
| **CD** | 0.70 (0.48-1.02) | 1.75 (1.40-2.19) |
| **UC** | 0.78 (0.54-1.13) | 2.08 (1.65-2.61) |
| IBD therapy group |  |  |
| **Steroids** | 0.82 (0.58-1.17) | 2.06 (1.64-2.58) |
| **Immunosuppressant** | 0.76 (0.48-1.18) | 1.69 (1.30-2.21) |
| **Any IBD therapy** | 0.78 (0.57-1.07) | 1.89 (1.54-2.32) |
| **Untreated for IBD** | 0.65 (0.40-1.04) | - 1. 1.45-2.43) |
| ***Adjusted for age at baseline, hypertension, hyperlipidemia, diabetes mellitus and BMI.** | | |

**Figure S1: Study population selection process**

Total

N=518,655

Unexposed

N=499,744

Exposed

N=18,911

Exclusion of:
1. Age<18 and >95 (n=56,802)
2. FU<180 days (n=23,505)
3. Prior diagnosis of CVD (n=16,175)
4. Dx of CKD/Hypercoagulability (n=12,451)

Total

N=431,364

Cutting exposed and unexposed groups into 10 age groups and matching individuals in a 1:10 ratio.

Unexposed

N=180,050

Exposed

N=18,005

Total

N=198,055

Exclusion of patients treated with aspirin/statins (as patient with higher average risk for CVD)

Total
N=135,106

**Figure S2: Crude HR of CVD among different IBD subgroups and therapy**


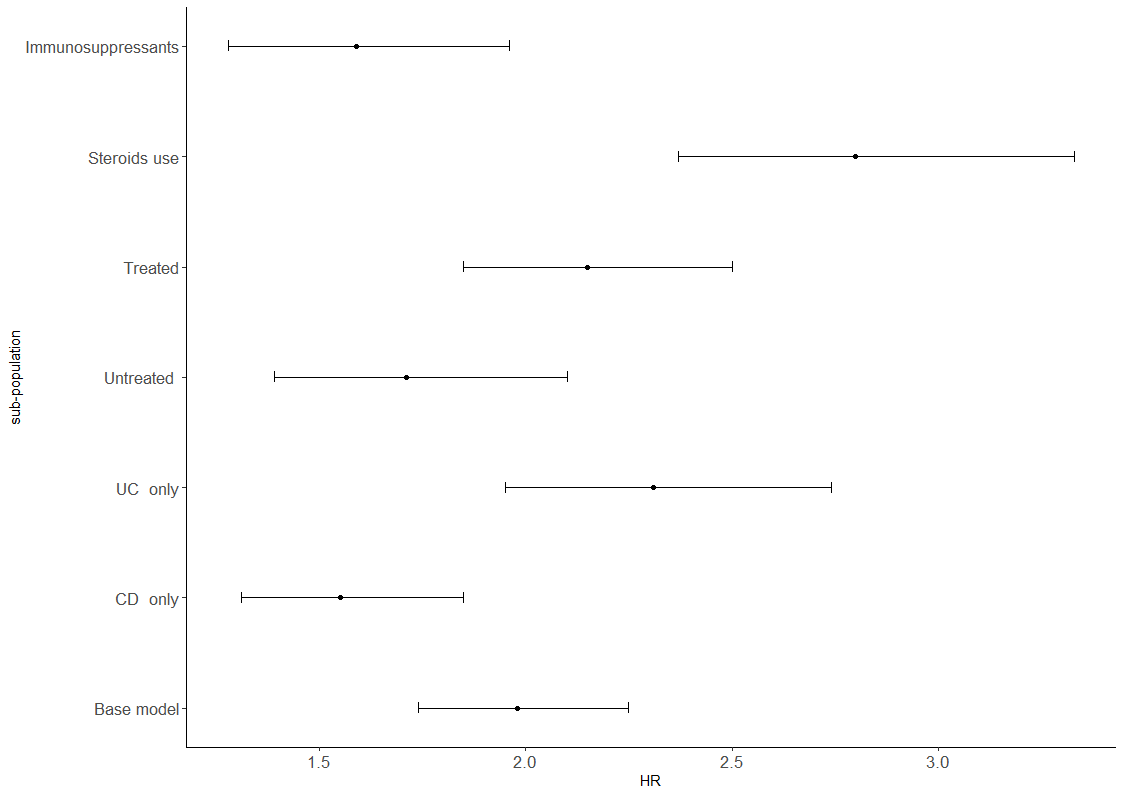


IBD subgroups and therapy

Hazard ratio

*Horizontal lines and central dots stand for crude HR and 95% confidence intervals.

**Figure S2: Age-and-sex specific Kaplan-Meier curves and their 95% confidence intervals of new onset of ischemic heart disease for patients with inflammatory disease (blue) and reference group (yellow). The P values are for the Log-Rank test.**


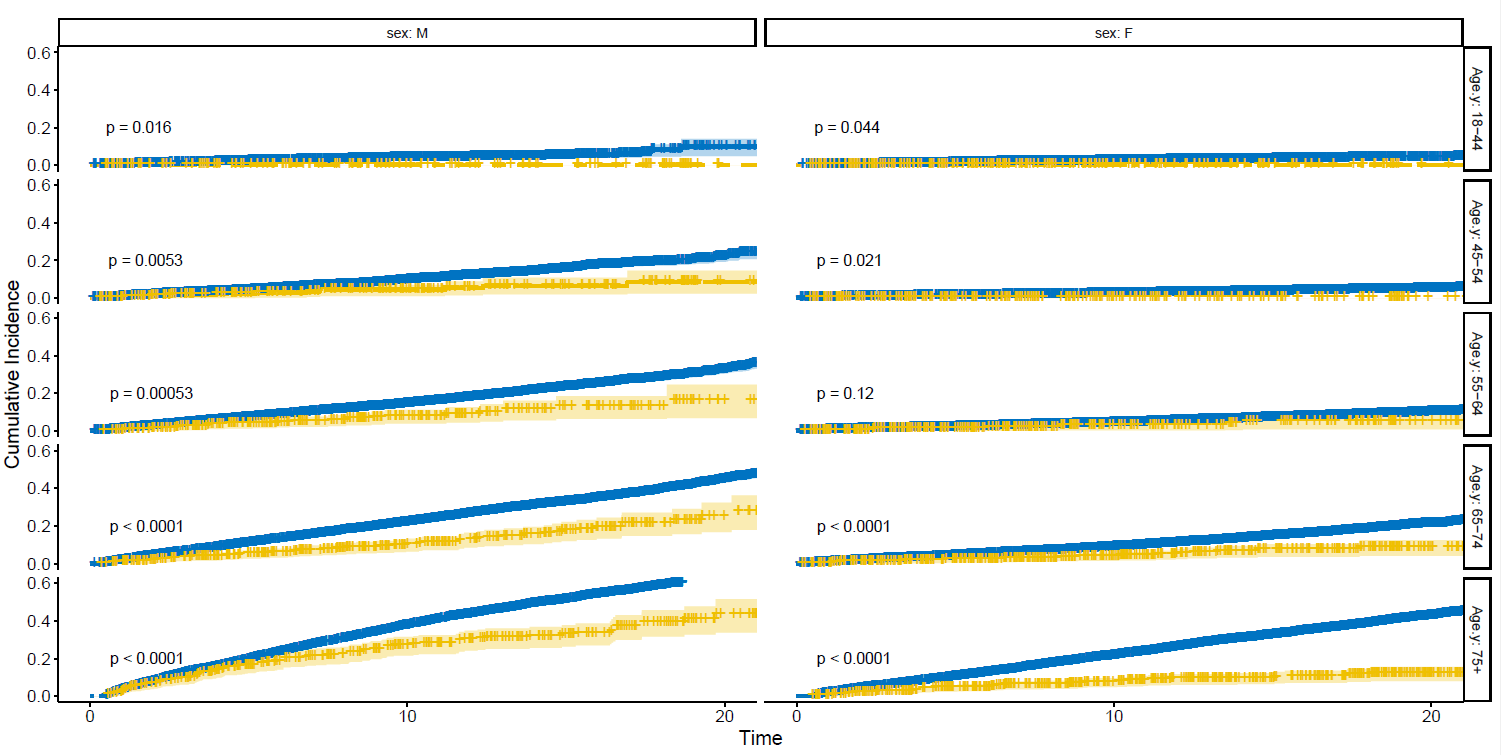


*Sex F=females, Sex M=males.
